# Proficiency in pole handling during Nordic walking influences exercise effectiveness in middle-aged and older adults

**DOI:** 10.1371/journal.pone.0208070

**Published:** 2018-11-27

**Authors:** Eiji Fujita, Karen Yakushi, Masaki Takeda, Mohammod Monirul Islam, Masaki Nakagaichi, Dennis Robert Taaffe, Nobuo Takeshima

**Affiliations:** 1 Department of Sports and Life Science, National Institute of Fitness and Sports in Kanoya, 1 Shiromizucho, Kanoya, Kagoshima, Japan; 2 Graduate School of Physical Education, National Institute of Fitness and Sports in Kanoya, 1 Shiromizucho,Kanoya, Kagoshima, Japan; 3 Faculty of Health and Sports Science, Doshisha University, 1–3 Tataramiyakodani, Kyotanabe, Kyoto, Japan; 4 Exercise Medicine Research Institute, School of Medical and Health Sciences, Edith Cowan University, Joondalup, Australia; 5 Department of Health and Sports Sciences, School of Health Sciences, Asahi University, 1851 Hozumi, Mizuho, Gifu, Japan; University of Bourgogne France Comté, FRANCE

## Abstract

Nordic walking (NW) is a total body version of walking increasingly used as a health-promoting activity by middle-aged and older adults. The present study examined the relationship between force exerted through the pole and physiological response during NW. In this non-randomized exercise trial, 17 participants comprising 8 middle-aged and older recreationally trained Nordic walkers (NWrec: 63.7 ± 8.1 years) and 9 experienced NW instructors (NWinstr: 57.5 ± 7.8 years) underwent outdoor ordinary walking (OW) and NW bouts as fast as possible for 12 minutes. Walking distance, speed, heart rate (HR), energy expenditure (METs and J/kg/m) and upper and lower limb muscle activities using surface electromyogram (EMG) were assessed. A pole with a built-in load cell measured force applied to the pole with peak pole force, pole contact time, % of pole contact time with respect to the gait cycle, and pole impulse derived. We conducted two-way analysis of covariance adjusted for age and BMI. There was a significant group and walking type interaction for walking distance and speed (P = 0.04), METs (P < 0.01), and HR (P = 0.04) with higher values in the NWinstr group during NW than OW. As expected, upper limb EMG activities increased (P < 0.01) with NW in both groups. All pole force measures were significantly higher in NWinstr than NWrec (P ≤ 0.01). Change in walking distance and speed were correlated with pole peak force (r = 0.67, P < 0.01) and pole impulse (r = 0.63, P = 0.01). Similarly, change in METs was associated with peak pole force (r = 0.66, P < 0.01) and pole impulse (r = 0.56, P = 0.02). These results indicate that planting the pole on the ground more forcefully and for longer periods to derive a driving force in NW enhances the effectiveness of the exercise and potentially the health-derived benefits.

## Introduction

The aging of the population is progressing globally, and between 2015 and 2050 the proportion of the world's population over 60 years will nearly double from 12% to 22% [1]. Similarly, Japan also has a rapidly aging population with over 33 million adults aged 65 years and older, accounting for 26.7% of the total population in 2015 [2]. As a result, in 2013, social security expenditure in Japan exceeded 111 trillion yen (~US$1.04 trillion), reaching a record high [3]. Therefore, extending the healthy life expectancy so that people can maintain independent living standards has become a critical issue. To address this, physical exercise performed more than twice a week for 30 min or longer is recommended as a health promotion measure in Japan [4].

Nordic walking (NW) was initially designed for cross-country ski athlete’s training in summer [5] and since then it has gained popularity worldwide as a health-promoting activity [6]. By incorporating upper body muscle activity similar to that in cross-country skiing, NW incorporates a total body version of walking with a greater caloric expenditure and potentially enhanced physical fitness benefits [5]. Previous studies comparing physiological response under the same conditions as ordinary walking (OW) have reported that NW results in higher upper limb muscle activity [7, 8]. Although less muscle activity has been reported in the lower limbs [7], oxygen uptake (${\dot{V}O}_{2}$) and energy expenditure have been shown to be higher during NW than OW [8].

A systematic review conducted by Tschentscher et al. [9] concluded that NW is an effective health-promoting physical activity as performing NW for a sustained period of time led to a lower resting heart rate and blood pressure, improved physical fitness, increased maximum ${\dot{V}O}_{2}$, and improved quality of life among individuals with various illnesses. Previous studies have also reported improved upper and lower limb muscle strength and flexibility along with enhanced general endurance among healthy older adults [10, 11]. Moreover, NW is viewed as a compound exercise that may be as effective as both aerobic and resistance training [11].

Given that NW uses the motion of pushing the poles into the ground to propel the body forward, it is highly likely that the proficiency in handling the poles may affect the physiological response. Several previous studies have suggested that differences in pole handling proficiency may affect exercise intensity and the effectiveness of NW [5, 12–15]. The International Nordic Walking Federation has established that the correct technique in use of the poles is active and dynamic, and control of the poles with the grip and strap to propel the body forward [16]. Moreover, Pellegrini et al. [12] reported that in male licensed NW instructors, NW shows a major pendulum-like energy recovery compared to OW which derived from the greater dynamic motion of the body’s center of mass and the swing of the arms and poles. However, no previous studies have conducted measurements undertaken outside the laboratory examining force exerted through the pole (an acquired pole handling skill) and physiological response during NW in Nordic walkers with difference experience/skill levels. Therefore, the present study examined the relationship between force exerted through the pole and physiological response during NW among middle-aged and older adults who regularly engaged in NW as a health-promoting exercise and more skillful NW instructors. We hypothesized that physiological responses (as a marker of potential health-derived benefits) would be greater in Nordic walkers with enhanced force exerted through the pole (the NW instructors).

## Materials and methods

### Study participants

Seventeen men and women comprising 8 community-dwelling middle-aged and older adults who had previously participated in a 9-week Nordic walking program and continued to engage in regular NW exercise (NWrec group: 2 men and 6 women), and 9 middle-aged licensed NW instructors from the Japan Nordic Fitness Association (NWinstr group: 3 men and 6 women) served as participants in the study. NWrec had been performing NW regularly twice a week, with each session lasting 40-60min, for 13 months. The NW instructors had 5 years of experience and had been performing NW regularly twice a week, with each session lasting 30–70 min. Both groups were apparently healthy and had no health condition that would adversely affect exercise capacity. This study was approved by the Ethics Committee of the National Institute of Fitness and Sports in KANOYA, Japan, and all participants provided written informed consent.

### Experimental protocol

**Fig 1:**
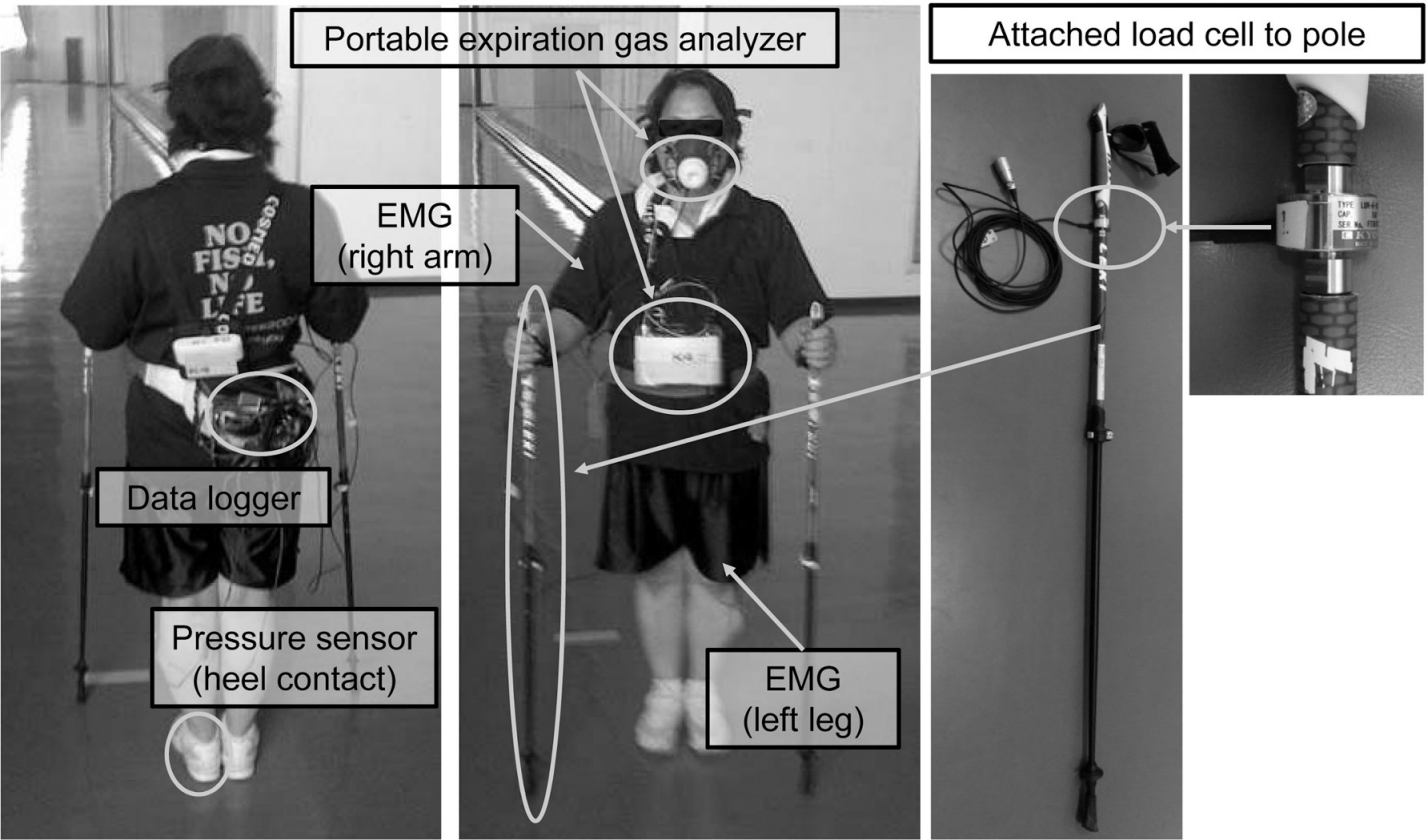
A participant wearing all the measurement devices. The EMGs signals and pole force signals were recorded via a same portable data logger. EMG: electromyogram.

The test session consisted of 12-min bouts of outdoor OW and NW using the diagonal technique (DIA) performed as fast as possible on a level asphalt surface (0% slope). The 12-min walking test has high reproducibility and validity for evaluating the cardiorespiratory fitness of the elderly [17]. DIA in NW is a walking technique where the pole held on the opposite side of the stepping foot is planted diagonally backwards [18]. The pole length was determined by multiplying each subject’s height in centimeters by 0.68. The order of OW and NW trials were carried out randomly by throwing a dice with a 20-min break between trials. The walking distance for both OW and NW trials were measured in 10-meter increments using a wrist-based optical HR monitor and portable global positioning system, with walking speed (m/min) calculated based on the obtained walking distance. Additionally, gait cycle frequency was measured in the last 1 min of each 12-min exercise trial. Heart rate (HR) was measured using the wrist-based optical HR monitor and a portable gas analyzer was used to assessed energy expenditure. Surface electromyography (EMG) was used to measure the muscle activity of the upper and lower limbs. A pole with a built-in load cell measured force used to push the pole into the ground (peak pole force), pole contact time, percentage of pole contact time with respect to duration of gait cycle (% pole contact time), and pole impulse (Fig 1). In the present study, peak pole force, pole contact time, % pole contact time, and pole impulse were used as indicators of pole proficiency. Throughout the study, the same technician conducted all data collection and analysis.

### Measurement of heart rate

A wrist-based optical HR monitor and portable global positioning system (M200; Polar Electro Oy, Kempele, Finland) was used to monitor HR, recorded at a sampling frequency of 1 Hz, with the mean HR during the last 1 min of the 12-min exercise trial reported. The wrist-based optical HR monitor with photoplethysmography technique is easy to wear for the elderly and provides an accurate measurement of HR during moderate intensity activities [19, 20].

### Measurement of metabolic responses

Metabolic responses which included energy expenditure were assessed using a portable gas analyzer (K4b2; COSMED Co. Ltd., Rome, Italy), with oxygen consumption in the last 1 min of each 12-min exercise trial. The metabolic measurements data were expressed in $\dot{V}E$ (l/min), ${\dot{V}O}_{2}$ (ml/min), ${\dot{V}\mathrm{CO}}_{2}$ (ml/min), respiratory exchange ratio (RER), metabolic equivalents (METs, where 1 MET = 3.5 ml O_2_/kg/min) and also the energy cost of walking (J/kg/m). Energy expenditure expressed in Joules (J) was calculated by obtaining the calorific value (Kcal) per 1000 ml oxygen from RER [21].

### Measurement of upper and lower limb muscle activity

Muscle activity of the right upper limb and left lower limb were measured with EMG during the exercise trial. The EMG was recorded from the right biceps brachii muscle (long head) and triceps brachii muscle (lateral head), and the left vastus lateralis muscle, biceps femoris muscle (long head), tibialis anterior muscle, and gastrocnemius muscle (lateral head) by bipolar configuration during an isometric maximal voluntary contraction (MVC) task and the exercise trial. Ag-Agcl electrodes (Blue Sensor N-00-S/25, Ambu A/S, Ballerup, Denmark) were placed over each muscle belly with an inter-electrode distance of 2 cm after the skin surface was cleaned with alcohol and rubbed with sandpaper. Electrodes were connected to a preamp-type EMG sensor (DL-141; S&ME Co. Ltd., Tokyo, Japan) with a bandwidth of 5-500Hz. Additionally, the timing of the heel contact, which was used to determine duration (sec) and frequency (times/min) of the gait cycle, was recorded by a pressure sensor (DL-250; S&ME Co. Ltd., Tokyo, Japan) attached to the left heel. The measurements were recorded at a sampling frequency of 1 KHz using a portable data logger (BioLog DL-2000; S&ME Co. Ltd., Tokyo, Japan) worn around the waist of each subject (Fig 1). The portable data logger is small and light, and weighs 170 g. The EMG sensors and its cords were covered with foam under-wrap tape to prevent artifacts in the EMG signals from T-shirt sleeves and shorts.

Initially, the maximum EMG activity was recorded for each muscle assessed by evoking the MVC (EMG_MVC_). According to the manual muscle testing method described by Daniels et al. [22], the biceps brachii muscle was measured during elbow flexion and the triceps brachii muscle during elbow extension against manual resistance. The EMG_MVC_ of the vastus lateralis muscle was measured using a custom-made dynamometer (T.K.K.5715; Takei Scientific Instruments Co. Ltd., Niigata, Japan), according to measurement procedures previously described [23]. The EMG_MVC_ of the tibialis anterior muscle and gastrocnemius muscle were measured using a custom-made dynamometer (D-08011C; Takei Scientific Instruments Co. Ltd., Niigata, Japan), in accordance with procedures previously described [24].

Prior to maximal efforts which were undertaken twice, participants undertook several submaximal practice exertions. The subjects were asked to exert ramp contractions to exert full strength within 5 s. Of the two MVC trials, the value with a visually higher EMG_MVC_ was selected. The EMG signals were full-wave rectified and integrated using analysis software (Chart 8.0; AD Instruments Ltd., Victoria, Australia). The EMG_MVC_ data during the middle 1 s of maximal EMG (3 s) were analyzed for each muscle as averaged rectified value (EMG_max_).

**Fig 2:**
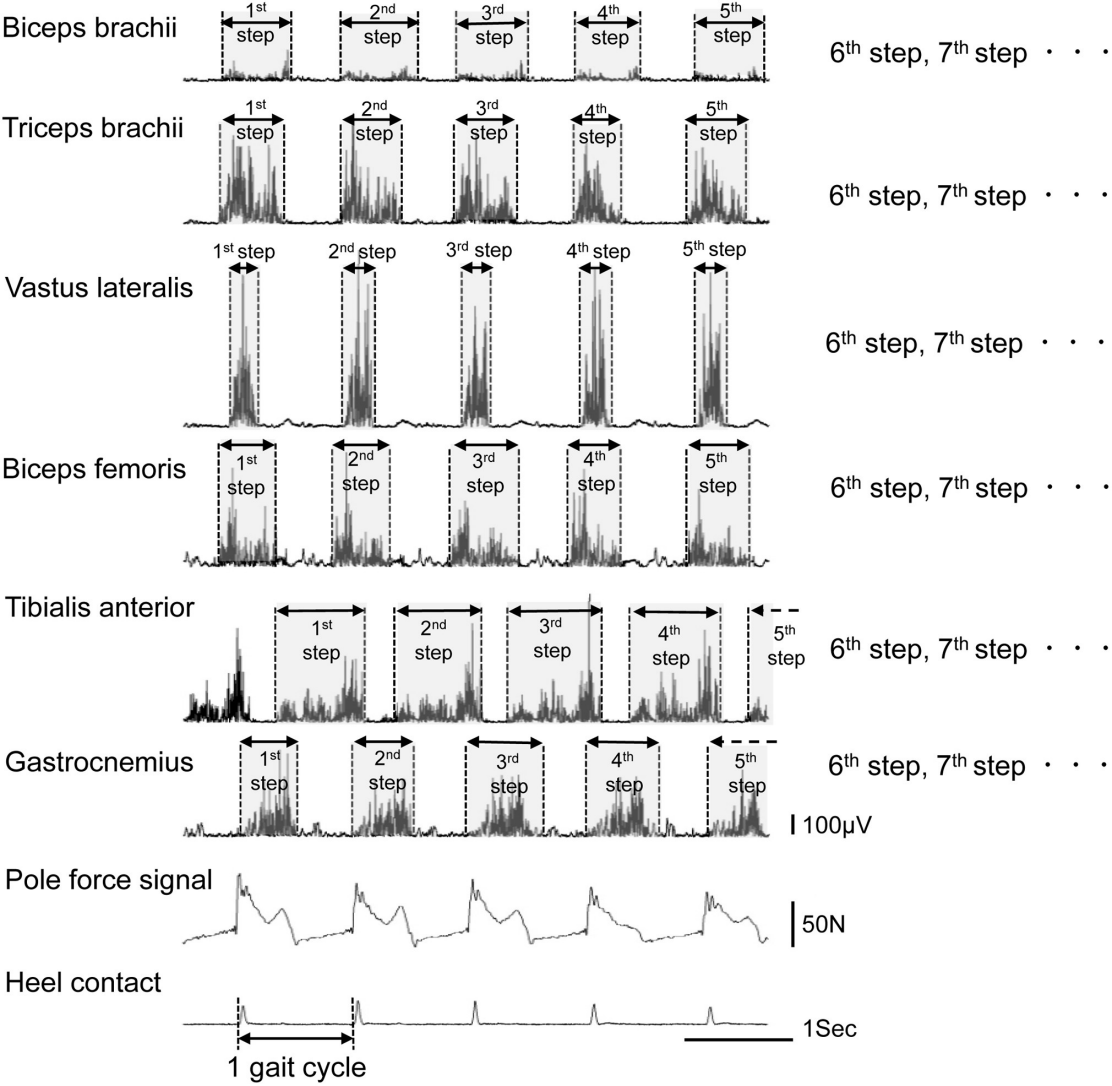
A typical example of the EMGs, pole force signal, and heel contact signal of a NWinstr participant during Nordic walking. The rectified EMG signals of each muscle during the walking task were averaged and normalized as the relative value to that during the MVC. EMG: electromyography, MVC: isometric maximal voluntary contraction.

The EMG data for the OW and NW trials were full-wave rectified and integrated as with the EMG_MVC_. The EMG data during the trials were analyzed for the most stable signal with the least noise for 10 continuous steps during the last minute of the 12-minute bout. Firstly, the muscle activity time for each step was determined from the EMG data of each muscle (Fig 2). Secondly, all integrated EMG data during the trials were divided by the specified time to find the averaged rectified values. Lastly, these averaged rectified values were normalized as the relative values to that during the MVC trials and averaged (%EMG_max_). The %EMG_max_ values for each muscle was used as an index representing the level of muscular activity of the upper and lower limb during OW and NW.

### Measurement of pole force signals

**Fig 3:**
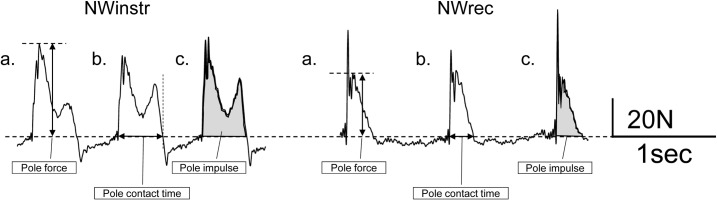
A typical example of the peak pole force, pole contact time and pole impulse data of NWinstr and NWrec participants during Nordic walking. (a) peak pole force, (b) pole contact time, (c) pole impulse. The calculation of peak pole force avoided the first peak force which is the value at the time of ground-contact and made it the second peak value. NW: Nordic walking, NWinstr: NW instructor, NWrec; recreationally trained Nordic walkers.

The measurement of pole force signals during the NW trial was conducted using Nordic poles with a built-in tension/compression load cell (LUR-A-1KNSA1; Kyowa Electronic Instruments Co. Ltd., Tokyo, Japan). Pole force signals data were amplified using a strain amplifier (DL-170A; S&ME Co. Ltd. Tokyo, Japan). The pole force signals were recorded via a portable data logger which was the same as that for EMG data collection (BioLog DL-2000; S&ME Co. Ltd. Tokyo, Japan) at a sampling frequency of 1 KHz worn around the waist of the subject. The pole force signals were recorded perfectly synchronous with the EMGs signals. Data were used to calculate the maximum force exerted on the long axis direction of the pole (peak pole force: N), the pole contact time (s), the percentage of pole contact time with respect to the gait cycle (%), and the pole impulse (Ns). The pole force signal data for the NW trial were analyzed for the same time periods as the EMG. An example of the peak pole force, pole contact time, and pole impulse data are shown in Fig 3.

### Statistical analyses

Statistical analyses were conducted using SPSS (version 25.0 for windows, SPSS Inc., Tokyo, Japan). Normality of the distribution for independent variables was tested using the Kolmogorov-Smirnov test. Between-group differences in participant characteristics (age, height, body weight, BMI) were assessed using unpaired Student’s t-tests or the Mann-Whitney U test as appropriate with effect size (ES) based on Cohen’s d and defined as small (d = 0.2), moderate (d = 0.5) and large (d ≥ 0.8). A two-way analysis of covariance (ANCOVA) was used to determine the effects of group (NWrec vs. NWinstr) and walking type (OW vs. NW), and their interaction (group-walking type) adjusted for BMI and age (age was added as a covariate due to the apparent difference between groups). If the two-way ANCOVA showed significant interaction, simple main effects tests were employed for individual comparisons with partial eta-square values provided as an indicator of ES. Data that were not normally distributed were log transformed for analysis. Differences in peak pole force, pole contact time, % pole contact time, and pole impulse were determined using unpaired Student’s t-test with ES (Cohen’s d) reported. The relationship between change in walking distance, speed, METs, energy cost of walking, and the sum of whole body, upper limb and lower limb muscle activity level (sum of whole body %EMG_max_, sum of upper limb %EMG_max_ and sum of lower limb %EMG_max_) with peak pole force, pole contact time, % pole contact time, and pole impulse were analyzed using the Pearson’s correlation coefficient. Pearson’s correlation was also used to examine the relationship between changes in METs, energy cost of walking and each sum of %EMG_max_. Changes in walking distance and speed, METs, energy cost of walking, and each sum of %EMG_max_ were obtained by the following formula: (NW value–OW value)/OW value × 100. Tests were two-tailed with level of significance set at P < 0.05. Values are reported as the mean ± SD with the 95% confidence interval (CI) also reported.

## Results

Participant characteristics are shown in Table 1. There was no significant difference in age, height, and mass between groups, although BMI was higher in NWrec (P = 0.01, ES: 1.40).

**Table 1:** Participant characteristics.

	NWrec	NWinstr	P-value	ES
Age (yr)	63.7 ± 8.1 (58.1–69.3)	57.5 ± 7.8 (52.4–62.6)	0.13	0.78
Height (cm)	154.5 ± 11.2 (146.8–162.2)	164.4 ± 8.9 (158.6–170.3)	0.06	0.99
Body mass (kg)	60.6 ± 11.9 (52.4–68.8)	57.4 ± 13.1 (48.8–65.9)	0.61	0.25
BMI (kg/m^2^)	25.2 ± 2.1 (23.7–26.6)	21.1 ± 3.5 (18.7–23.4)	0.01	1.40

Values are mean ± SD (95% CI), NWrec: recreationally trained Nordic walkers, NWinstr: Nordic walking instructors, ES: effect size (Cohen’s d), BMI: body mass index.

### Walking distance and speed

The walking distances of the NWrec group were 1015.0 ± 84.0 m and 1081.3 ± 89.5 m during the OW and NW sessions, respectively, with a mean walking speed of 84.6 ± 7.0 m/min and 90.1 ± 7.5 m/min. In comparison, the walking distance of the NWinstr group were 1140.0 ± 109.4 m and 1315.6 ± 159.2 m during the OW and NW sessions, respectively, and therefore mean walking speed increased from 95.0 ± 9.1 m/min to 109.6 ± 13.3 m/min. Results of the two-way ANCOVA indicated that there was a significant interaction between group and walking type (walking distance: F = 8.12, P = 0.01, walking speed: F = 8.13, P = 0.01). Analysis of simple main effects indicated that walking type and group were significantly different (OW: NWrec < NWinstr, NW: NWrec < NWinstr, NWinstr: OW < NW) (Table 2). Gait cycle frequency in the last 1 min of each 12-min exercise trial in the NWrec group was 64.9 ± 4.5 times/min and 63.6 ± 3.9 times/min during the OW and NW sessions, respectively. In comparison, gait cycle frequency in the NWinst was 62.7 ± 3.3 times/min and 62.9 ± 3.8 times/min during the OW and NW sessions, respectively. There was no significant interaction between group and walking type (F = 1.08, P = 0.32), nor was there a significant main effect for group (F = 0.45, P = 0.51) or walking type (F = 0.49, P = 0.49) (Table 2).

**Table 2:** Result of walking distance and speed, and heart rate.

	Walking type	NWrec	NWinstr	Main effect		Interaction		ES
				Group	Walking type	Group × Walking type		
				*F* value (P value)		*F* value (P value)	Simple main effect	
Walking distance (m)	OW	1015.0 ± 84.0	1140.0 ± 109.4	*F* = 10.27 (P = 0.01)	*F* = 36.93 (P < 0.01)	*F* = 8.12 (P = 0.01)	OW: NWrec < NWinstr NW: NWrec < NWinstr NWinstr: OW < NW	0.39
		(956.8–1073.2)	(1068.5–1211.5)					
	NW	1081.3 ± 89.5	1315.6 ± 159.2					
		(1019.2–1143.3)	(1211.5–1419.6)					
Walking speed (m/min)	OW	84.6 ± 7.0	95.0 ± 9.1	*F* = 10.27 (P = 0.01)	*F* = 36.94 (P < 0.01)	*F* = 8.13 (P = 0.01)	OW: NWrec < NWinstr NW: NWrec < NWinstr NWinstr: OW < NW	0.39
		(79.7–89.4)	(89.0–101.0)					
	NW	90.1 ± 7.5	109.6 ± 13.3					
		(84.9–95.3)	(101.0–118.3)					
Gait cycle (times/min)	OW	64.9 ± 4.5	62.7 ± 3.3	*F* = 0.45 (P = 0.51)	*F* = 0.49 (P = 0.49)	*F* = 1.08 (P = 0.32)		0.08
		(61.7–68.0)	(60.5–64.8)					
	NW	63.6 ± 3.9	62.9 ± 3.8					
		(60.9–66.3)	(60.4–65.3)					
Heart rate (bpm)	OW	130 ± 18	114 ± 13	*F* = 0.14 (P = 0.72)	*F* = 14.62 (P < 0.01)	*F* = 5.45 (P = 0.04)	NWinstr: OW < NW	0.30
		(116.8–142.5)	(105.3–122.8)					
	NW	132 ± 20	135 ± 19					
		(118.5–146.1)	(122.7–148.1)					

Values are mean ± SD (95%CI), NWrec: recreationally trained Nordic walkers, NWinstr: Nordic walking instructors, ES: effect size (partial η^2^), OW: ordinary walking, NW: Nordic walking.

### Heart rate

HR in NWrec group was 130 ± 18 bpm and 132 ± 20 bpm during the OW and NW sessions, respectively. In comparison, HR in the NWinstr group was 114 ± 13 bpm and 135 ± 19 bpm during the OW and NW sessions, respectively. Results of the two-way ANCOVA showed that there was significant interaction between group and walking type (F = 5.45, P = 0.04). Analysis of simple main effects indicated that group was significantly different with HR significantly greater during NW than during OW in NWinstr (Table 2).

### Metabolic responses

For $\dot{V}E$, ${\dot{V}O}_{2}$, and ${\dot{V}\mathrm{CO}}_{2}$, there were significant group by walking type interactions ($\dot{V}E$: F = 9.52, P < 0.01; ${\dot{V}O}_{2}$: 18.26, P < 0.01; ${\dot{V}\mathrm{CO}}_{2}$: F = 12.31, P < 0.01). Analysis of simple main effects indicated that walking type and group were significantly different in the $\dot{V}E$, ${\dot{V}O}_{2}$ (NW: NWrec < NWinstr, NWinstr: OW < NW) and ${\dot{V}\mathrm{CO}}_{2}$ (OW: NWrec < NWinstr, NW: NWrec < NWinstr, NWinstr: OW < NW) (Table 3).

**Table 3:** Result of metabolic measurements.

	Walking type	NWrec	NWinstr	Main effect		Interaction		ES
				Group	Walking type	Group × Walking type		
				*F* value (P value)		*F* value (P value)	Simple main effect	
$\dot{V}E$ (l/min)	OW	35.4 ± 12.0	37.8 ± 8.7	*F* = 4.68 (P = 0.05)	*F* = 17.40 (P < 0.01)	*F* = 9.52 (P = 0.01)	NW: NWrec < NWinstr NWinstr: OW < NW	0.42
		(27.0–43.7)	(32.1–43.5)					
	NW	39.9 ± 17.6	52.5 ± 16.3					
		(27.7–52.0)	(41.8–63.1)					
${\dot{V}O}_{2}$ (ml/min)	OW	1140.6 ± 360.5	1181.1 ± 288.0	*F* = 4.24 (P = 0.06)	*F* = 25.48 (P < 0.01)	*F* = 18.26 (P < 0.01)	NW: NWrec < NWinstr NWinstr: OW < NW	0.58
		(890.8–1390.4)	(993.0–1369.3)					
	NW	1176.5 ± 371.8	1542.5 ± 341.8					
		(918.8–1434.1)	(1319.2–1765.8)					
${\dot{V}\mathrm{CO}}_{2}$ (ml/min)	OW	1105.9 ± 357.3	1332.8 ± 305.7	*F* = 6.73 (P = 0.02)	*F* = 33.49 (P < 0.01)	*F* = 12.31 (P < 0.01)	OW: NWrec < NWinstr NW: NWrec < NWinstr NWinstr: OW < NW	0.49
		(858.2–1353.5)	(1133.1–1532.5)					
	NW	1351.4 ± 519.5	1838.3 ± 545.0					
		(991.5–1711.4)	(1482.3–2194.4)					
RER	OW	0.967 ± 0.029	1.143 ± 0.147	*F* = 4.48 (P = 0.05)	*F* = 6.44 (P = 0.03)	*F* = 0.05 (P = 0.83)		0.00
		(0.947–0.987)	(1.047–1.239)					
	NW	1.142 ± 0.150	1.183 ± 0.117					
		(1.039–1.246)	(1.107–1.260)					
METs	OW	5.4 ± 1.1	5.5 ± 0.9	*F* = 4.07 (P = 0.07)	*F* = 25.59 (P < 0.01)	*F* = 14.74 (P < 0.01)	NW: NWrec < NWinstr NWinstr: OW < NW	0.53
		(4.6–6.1)	(5.0–6.1)					
	NW	5.5 ± 1.2	7.3 ± 1.1					
		(4.7–6.4)	(6.5–8.0)					
Energy cost of walking (J/kg/m)	OW	4.6 ± 0.9	4.3 ± 0.8	*F* = 0.17 (P = 0.69^#^)	*F* = 3.36 (P = 0.09^#^)	*F* = 4.24 (P = 0.06^#^)		0.25
		(4.0–5.3)	(3.8–4.8)					
	NW	4.5 ± 0.9	4.9 ± 0.5					
		(3.9–5.2)	(4.6–5.2)					

Values are mean ± SD (95%CI), NWrec: recreationally trained Nordic walkers, NWinstr: Nordic walking instructors, ES: effect size (partial η^2^), OW: ordinary walking, NW: Nordic walking, RER: respiratory exchange ratio

#: P-value based on log-transformed data.

For the RER, there was no significant group by walking type interaction (F = 0.05, P = 0.83), however, there was significant main effect of walking type (F = 6.44, P = 0.03) (Table 3).

For METs, there was a significant interaction between group and walking type (F = 14.74, P < 0.01). Analysis of simple main effects indicated that walking type and group were significantly different (NW: NWrec < NWinstr, NWinstr: OW < NW) (Table 3).

There was no significant interaction for the energy cost of walking (F = 4.24, P = 0.06). Also, there was no significant main effect for group (F = 0.17, P = 0.69) or walking type (F = 3.36, P = 0.09) (Table 3).

### Muscle activity levels

Biceps brachii muscle activity level was 2.4 ± 1.4%EMG_max_ and 8.0 ± 5.3%EMG_max_ during the OW and NW sessions in the NWrec group, respectively, and 2.0 ± 1.6%EMG_max_ and 8.3 ± 4.7%EMG_max_ in the NWinstr group. There was no significant interaction between group and walking type (F = 0.03, P = 0.88), however, there was a significant main effect for walking type (F = 85.22, P < 0.01) with muscle activity greater during NW than OW (Table 4). Similarly, for triceps brachii muscle activity there was no significant interaction between group and walking type (F = 0.02, P = 0.90) but a significant main effect of walking type (F = 30.12, P < 0.01).

**Table 4:** Result of muscle activity levels.

	Walking type	NWrec	NWinstr	Main effect		Interaction		ES
				Group	Walking type	Group × Walking type		
				*F* value (P value)		*F* value (P value)	Simple main effect	
Biceps Brachii (%)	OW	2.4 ± 1.4	2.0 ± 1.6	*F* = 0.16 (P = 0.69)	*F* = 85.22 (P < 0.01)	*F* = 0.03 (P = 0.88)		0.00
		(1.4–3.4)	(0.9–3.0)					
	NW	8.0 ± 5.3	8.3 ± 4.7					
		(4.3–11.7)	(5.2–11.3)					
Triceps brachii (%)	OW	2.3 ± 2.7	5.2 ± 3.7	*F* = 1.02 (P = 0.33^#^)	*F* = 30.12 (P < 0.01^#^)	*F* = 0.02 (P = 0.90^#^)		0.00
		(0.4–4.2)	(2.8–7.6)					
	NW	19.7 ± 13.7	22.2 ± 9.8					
		(10.2–29.2)	(15.8–28.6)					
Vastus lateralis (%)	OW	31.7 ± 16.6	24.7 ± 11.4	*F* = 0.06 (P = 0.81^#^)	*F* = 4.99 (P = 0.04^#^)	*F* = 0.82 (P = 0.38^#^)		0.06
		(20.2–43.2)	(17.3–32.2)					
	NW	26.8 ± 24.5	21.6 ± 8.9					
		(9.9–43.8)	(15.8–27.3)					
Biceps femoris (%)	OW	17.3 ± 7.9	15.4 ± 8.1	*F* = 0.11 (P = 0.75^#^)	*F* = 0.21 (P = 0.65^#^)	*F* = 0.07 (P = 0.80^#^)		0.01
		(11.8–22.8)	(10.1–20.6)					
	NW	16.4 ± 6.8	15.2 ± 7.9					
		(11.7–21.1)	(10.0–20.3)					
Tibialis anterior (%)	OW	35.1 ± 15.2	21.5 ± 11.4	*F* = 2.57 (P = 0.13^#^)	*F* = 0.01 (P = 0.93^#^)	*F* = 7.36 (P = 0.02^#^)	OW: NWrec > NWinstr	0.36
		(24.6–45.6)	(14.1–28.9)					
	NW	30.0 ± 13.0	25.3 ± 14.2					
		(21.0–39.0)	(16.1–34.6)					
Gastro (%)	OW	69.8 ± 22.1	49.0 ± 15.5	*F* = 6.227 (P = 0.03)	*F* = 8.26 (P = 0.01)	*F* = 0.01 (P = 0.96)		0.00
		(54.4–85.1)	(38.9–59.1)					
	NW	54.9 ± 25.9	38.6 ± 20.9					
		(37.0–72.9)	(24.9–52.2)					
Sum of upper limb %EMG_max_ (%)	OW	4.7 ± 3.6	7.2 ± 3.1	*F* = 3.35 (P = 0.09^#^)	*F* = 85.16 (P < 0.01^#^)	*F* = 1.16 (P = 0.30^#^)		0.08
		(2.2–7.2)	(5.2–9.2)					
	NW	27.7 ± 18.6	30.5 ± 10.1					
		(14.8–40.6)	(23.9–37.1)					
Sum of lower limb %EMG_max_ (%)	OW	153.8 ± 49.1	110.6 ± 24.0	*F* = 3.66 (P = 0.08)	*F* = 6.69 (P = 0.02)	*F* = 0.59 (P = 0.46)		0.04
		(119.8–187.9)	(94.9–126.3)					
	NW	128.1 ± 50.9	100.6 ± 28.7					
		(92.8–163.4)	(81.9–119.4)					
Sum of Whole body %EMG_max_ (%)	OW	158.5 ± 48.3	117.8 ± 23.6	*F* = 2.28 (P = 0.16)	*F* = 0.49 (P = 0.50)	*F* = 0.41 (P = 0.53)		0.03
		(125.1–192.0)	(102.4–133.2)					
	NW	155.8 ± 64.0	131.1 ± 36.5					
		(111.5–200.2)	(107.3–154.9)					

Values are mean ± SD (95%CI), NWrec: recreationally trained Nordic walkers, NWinstr: Nordic walking instructors, ES: effect size (partial η^2^), OW: ordinary walking, NW: Nordic walking, Gastro: Gastrocnemius

#: P-value based on log-transformed data.

In comparison, for lower limb muscle activity level, there was a significant interaction between group and walking type in the tibialis anterior muscle (F = 7.34, P = 0.02). Analysis of simple main effects indicated that walking type was significantly different (OW: NWrec > NWinstr). For other lower limb muscle activity levels, there were significant main effects for group (gastrocnemius: F = 6.23, P = 0.03) and walking type (vastus lateralis: F = 4.99, P = 0.04; gastrocnemius: F = 8.26, P = 0.01) (Table 4).

For the sum of upper limb %EMG_max_, there was no significant interaction (F = 1.16, P = 0.30), however, there was a significant main effect of walking type (F = 85.16, P < 0.01) with the sum of upper limb muscle activity greater during NW than OW. Also, in the sum of lower limb %EMG_max_, there was no significant interaction (F = 0.59, P = 0.46), but a significant main effect of walking type (F = 6.69, P = 0.02) with the sum of lower limb muscle activity less during NW than OW (Table 4). In comparison, in the sum of whole body %EMG_max_, there was no significant interaction (F = 0.41, P = 0.53), or main effects for group (F = 2.28, P = 0.16) and walking type (F = 0.49, P = 0.50) (Table 4).

### Poling force signals

**Fig 4:**
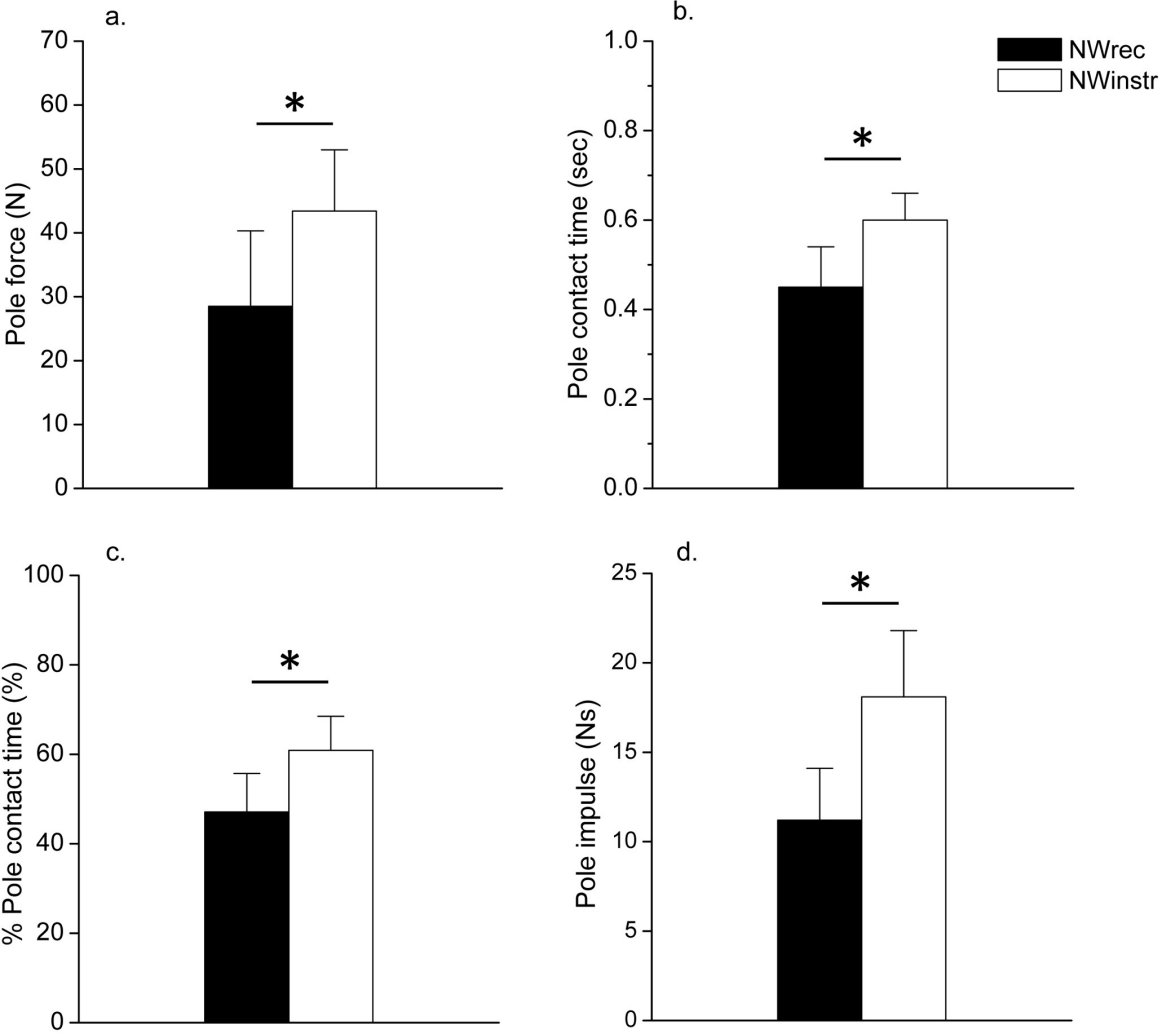
Comparison peak pole force, pole contact time, % pole contact time, and pole impulse between the NWrec group and the NWinstr group during Nordic walking. (a) peak pole force, (b) pole contact time, (c) % pole contact time, (d) pole impulse. NWrec: recreationally trained Nordic walkers, NWinstr: Nordic walking instructors, *:NWrec group VS. NWinstr group (P < 0.05).

Peak pole force during NW was significantly higher in the NWinstr than in NWrec (43.4 ± 9.6 N vs. 28.5 ± 11.8 N, P = 0.01, ES = 1.4) (Fig 4A). Similarly, pole contact time was longer in the NWinstr than NWrec (0.60 ± 0.06 sec vs. 0.45 ± 0.09 sec, P < 0.01, ES = 1.99) (Fig 4B), % pole contact time was higher in the NWinstr than NWrec (60.9 ± 7.6% vs. 47.1 ± 8.6%, P < 0.01, ES = 1.7) (Fig 4C), and pole impulse was greater (18.1 ± 3.7 Ns vs. 11.2 ± 2.9 Ns, P < 0.01, ES = 2.08) (Fig 4D).

Changes in walking distance and speed was significantly correlated with peak pole force (r = 0.67, P < 0.01) and pole impulse (r = 0.63, P = 0.01) (Table 5). Similarly, change in METs was significantly associated with peak pole force (r = 0.66, P < 0.01) and pole impulse (r = 0.56, P = 0.02) and the energy cost of walking was significantly correlated with % pole contact time (r = 0.51, P = 0.04). There were no significant correlations between each sum of %EMG_max_ and the pole force variables.

**Table 5:** Relationship between pole force variables with change in walking distance and speed, METs, energy cost of walking, and amount of %EMG_max_.

	Peak pole force	Pole contact time	% pole contact time	Pole impulse
Changes in walking distance and speed	r = 0.67, P < 0.01	r = 0.05, P = 0.86	r = 0.08, P = 0.76	r = 0.63, P = 0.01
Changes in METs	r = 0.66, P < 0.01	r = 0.38, P = 0.13	r = 0.47, P = 0.06	r = 0.56, P = 0.02
Changes in energy cost of walking	r = 0.41, P = 0.10	r = 0.43, P = 0.09	r = 0.51, P = 0.04	r = 0.32, P = 0.21
Changes in the sum of upper limb %EMG_max_	r = -0.21, P = 0.41	r = -0.42, P = 0.10	r = -0.41, P = 0.10	r = -0.31, P = 0.23
Changes in the sum of lower limb %EMG_max_	r = 0.22, P = 0.40	r = 0.18, P = 50	r = 0.39, P = 0.12	r = 0.25, P = 0.33
Changes in the sum of whole body %EMG_max_	r = 0.23, P = 0.28	r = 0.18, P = 0.50	r = 0.40, P = 0.12	r = 0.25, P = 0.33

### Relationship between change in METs and the energy cost of walking with each sum of %EMG_max_

Change in METs was significantly correlated with change in the energy cost of walking (r = 0.88, P < 0.01) and change in the sum of lower limb %EMG_max_ (r = 0.52, P = 0.03) and whole body %EMG_max_ (r = 0.66, P < 0.01). In addition, change in the energy cost of walking was significantly associated with change in the sum of lower limb %EMG_max_ (r = 0.57, P = 0.02) and whole body %EMG_max_ (r = 0.67, P < 0.01). However, there were no significant correlations between sum of upper limb %EMG_max_ and change in METs (r = -0.18, P = 0.50) and the energy cost of walking (r = -0.06, P = 0.82).

## Discussion

In this study, we examined the relationship between force exerted through the pole as an indicator of pole proficiency and physiological response during Nordic walking in middle-aged and older persons. There were two important findings: 1) peak pole force, pole contact time, % pole contact time, and pole impulse were significantly higher in NW instructors compared to recreationally trained Nordic walkers; and 2) change in walking distance, speed, and METs were significantly correlated with peak pole force and pole impulse. These findings suggest that in NW pushing the pole more forcefully and for longer periods on the ground results in increased walking distance, speed, and METs during exercise which may contribute to enhanced health-derived activity benefits, supporting our study hypothesis. These results are in agreement with those of Pellegrini et al. [15] who reported that a weak pole action (reducing the propulsive action exerted along the pole) decreased energy expenditure during NW compared to the correct pole handling technique. As such, those seeking to engage in NW for health-related benefits would benefit from appropriate instruction on pole handling so that potential benefits may be achieved.

The EMG results indicated that muscle activity in the upper limbs of both groups significantly increased with NW. As previously reported [8, 16, 18], muscle activity of the upper limbs, particularly for the triceps brachii muscles, inevitably increases in NW since participants use poles to push backwards in order to propel the body forward. In contrast, the vastus lateralis muscle and gastrocnemius muscle activities significantly decreased with NW. By the same token, the sum of lower limb %EMG_max_ significantly decreased with NW. It may well be that there was some compensatory reduction in lower limb muscle activity as upper limb activity increased in NW. However, previous reports are somewhat mixed regarding muscle activity of the lower limbs, with some reporting decreased activity [7, 19] and others no change [14]. Further research is required to clarify the contribution of lower limb muscle activity during NW.

Although there were no significant group differences in muscle activity of the upper limbs during NW, peak pole force was significantly higher in the NWinstr group than in the NWrec group. According to Hasegawa et al. [25], the peak pole force of college ski athletes performing NW on a level surface at a speed of 6 km/h was 16.5 N. This value is low compared with the present study. However, for the college ski athletes in the Hasegawa et al. [25] study, NW at a speed of 6 km/h was a low- to moderate-intensity exercise, and a weak peak pole force may be an understandable result compared with our study, which required our participants to walk as fast as possible. Moreover, NW in the study by Hasegawa et al. [25] was conducted on a treadmill, however, it has been pointed out the difficulty in effectively using poles as a driving force on the moving belt of a treadmill [26]. In contrast, Schiffer et al. [27] reported that peak pole force, pole contact time, and pole impulse of 36.5 N, 0.38 s, and 7.1 Ns, respectively, in 13 young adult female NW instructors through field measurements on a concrete surface which are somewhat similar to values in our study especially for NWrec. However, the values were lower than for our NWinstr group and this may be due to the faster walking speed in Schiffer et al. of 2.2 m/sec [27], given that the efficiency of propulsive force by the pole decreases as the speed of walking/running transition [26].

In the NWinstr group, peak pole force, pole contact time, % contact time, and pole impulse were significantly higher than in NWrec group. Moreover, peak pole force and pole impulse were significantly associated with change between OW and NW in walking distance, speed, and METs. These results suggest that NW is superior to OW as a health-promoting exercise, at least as far as METs is concerned in those who more forcefully push the pole to the ground to propel the body forward. It has been previously reported that the correct NW technique permitted an increase in energy expenditure during NW [5, 15], which is in agreement with our results. On the other hand, Schiffer et al. [27] reported that in the pole handling of licensed NW instructors, pole inclination starts from 70° at initial pole contact to 45° at terminal pole off during pole ground contact. As a result, pole impulses are applied in a horizontal direction between one third (at initial pole contact) and two thirds (terminal pole off) of the axial pole forces. This indicates that proficiency in handling the poles to derive a driving force is not only related merely to the push, but also elements such as which direction and timing to push the pole. Taking these factors into account, the enhanced proficiency in pole handling involves pushing the pole more forcefully with consideration for pole inclination to propel the body forward. In this study, walking distance and speed significantly increased with NW in the NWinstr group. Nonetheless, there was no significant difference between OW and NW in gait cycle frequency. These results suggest that stride length in the NWinstr group increased with NW, and the increased stride length seems to be due to the propulsion by correct pole handling. However, pole handling and walking motion analysis were not conducted in the current study.

A number of previous studies have shown that NW ensures higher exercise effectiveness compared with OW [7–9, 11, 15, 16, 18, 25, 28, 29]. However, in order to obtain a substantial exercise effect at NW, it is necessary to acquire correct pole handling and proper walking method [5, 14, 15, 26]. The present study used the force exerted on the long axis direction of the pole during NW (peak pole force), pole contact time, % pole contact time, and pole impulse as indicators of pole proficiency to compare the two group. The waveform of the pole force signals indicated that NWinstr group plant their poles on the ground more forcefully and for longer periods of time compared with NWrec, and this led to higher METs during exercise. By the same token, Pellegrini et al. [12] suggest that the amount of increase in energy expenditure when walking with poles is not a universal value which is likely explained by the pole handling technique.

A limitation of this study was that exercise intensity was not strictly controlled, such as can occur when using a treadmill, and participants were only instructed to “walk as fast as possible” over the 12-minute walking bout. Based on HR response during the exercise bouts it would appear that that NWrec were working at a higher percentage of what would be their predicted HRmax (220 –age) during OW than the NWinstr and, as a result, may have already reached or were close to their maximal physiological exertion capacity during the 12-minute walking bout. In contrast, it appears that the NW instructors were working at a lower percentage of their HRmax during OW and hence had more room for greater exertion during NW. The use of ratings of perceived exertion such as the Borg scale [30] during exercise was not undertaken but would have been a useful measure to ensure appropriate exercise exertion during the field-based test. Although this is a limitation, compared to previous studies that compared physiological responses between NW and OW undertaken on a treadmill [7, 8, 14, 15, 16, 18, 25, 28], a strength of the present study was that we did collect actual field measurements which we believe more closely replicates actual NW practice. Further, our data logger which was used to collect EMG, pole force and heel contact timing had an 8-channel input, and EMG signals could only record 6 muscles. Pellegrini et al. [16] reported that in NW there was greater muscle activity not only from the upper arm but also from the trunk (rectus abdominis, trapezius, latissimus dorsi) as well as the anterior deltoid muscle than during OW. Consequently, additional muscles to that investigated in the current study may have also contributed to the increased force during NW.

In conclusion, the present study found that planting the pole on the ground more forcefully and for longer periods to derive a driving force in NW increased walking distance, speed, and METs during exercise which may contribute to enhanced health-derived activity benefits in middle-aged and older adults. As a result, to maximize benefits from this activity mode participants undertaking Nordic walking should be provided with sufficient instruction on pole handling such as to push the pole forcefully and firmly extending the arm behind the body to propel the body forward, so that the health-promoting effects from this total body exercise may be optimized.
